# Glomerular diseases outcome at one year in a tertiary care centre

**DOI:** 10.12669/pjms.312.6779

**Published:** 2015

**Authors:** Huma Mamun Mahmud, Darshan Kumar, Humera Irum, Syed Farman Ali

**Affiliations:** 1 Dr. Huma Mamun Mahmud, MBBS, MCPS (Med), FCPS (Nephrol). Assistant Professor, Consultant Nephrologist, Department of Medicine, Dow International Medical College, Dow University of Heath Sciences, Karachi, Pakistan; 2 Dr. Darshan Kumar, MBBS, FCPS (Med). Assistant Professor, Dow International Medical College, Dow University of Heath Sciences, Karachi, Pakistan. Dow International Medical College (Ojha Campus), DUHS, Suparco Road, off Main University Road, Gulzar-e-Hijri, Scheme 33, Karachi, Pakistan; 3 Dr. Humera Irum, MBBS. Senior Medical Officer (Nephrology), Dow International Medical College, Dow University of Heath Sciences, Karachi, Pakistan. Dow International Medical College (Ojha Campus), DUHS, Suparco Road, off Main University Road, Gulzar-e-Hijri, Scheme 33, Karachi, Pakistan; 4 Dr. Syed Farman Ali, Dow International Medical College, Dow University of Heath Sciences, Karachi, Pakistan

**Keywords:** Glomerular diseases, Proteinuria, CKD, ESRF, Outcome

## Abstract

**Objectives:**

To determine outcome in primary and secondary glomerular diseases at one year follow up.

**Methods:**

Study design is observational cohort, done in out-patient department, Dow Iinternational Medical College, DUHS. All information gathered on a proforma. All patients with dipstick positive proteinuria and clinical glomerular disease were included in study. Patients with no proteinuria were excluded so were patients with stage 5 CKD. Patients were followed for proteinuria and renal insufficiency at completion of one year follow up. Statistical analysis was done on SPSS version 16.

**Result:**

Total number of patients who completed one year follow up was 173. Mean age of patients was 51.67+ 10.16 (range 15 to 75 years). Ninety two (53.2%), were males and 81(46.8%) were females, ratio being 1.1: 1.0. Mean weight of our patients was 67.43+ 14.13 Kg, (35 to 107 kg). Commonest cause of glomerular disease in our patient was diabetic nephropathy which was seen in 94.2% patients. Commonest associated problem with glomerular disease was hypertension seen in 66.5% of patients. Four out of 173 patients had stage 5 CKD at end of follow up at one year while quantitativ proteinuria remained same at one year follow up.

**Conclusion:**

One year follow up is critical for patients with glomerular disease associated with stage 4 CKD as progression to end stage renal failure may be seen within one year in these patients.

## INTRODUCTION

Glomerular diseases are an important cause of morbidity and mortality. With appropriate care the rate of progression of these diseases can be slowed down and can delay onset of end stage renal failure (ESRF). There are a number of locally published studies on glomerular diseases diagnosed on renal biopsy.^1^ Commonest primary glomerular disease among children is found focal and segmental glomerulosclerosis (FSGS)^2^ and primary glomerular diseases accounts for 49% and secondary glomerular diseases 30%.^3^ The commonest primary disease was membranoprolifeartive and commonest secondary disease was lupus in adults.^3^

Study from Oman has shown primary glomerular diseases in 58% and secondary glomerular diseases in 30.4% of 424 renal biopsies^4^, commonest secondary cause was lupus and primary was FSGS^4^. Most of this local and international data on glomerular disease frequency is from lab evaluation, being done through reported renal biopsies. Only few studies are published to comment on outcome of glomerular disease and follow up are not available from locally published studies. Diabetes, hypertension and dyslipidemia are factors which are very commonly seen in patients with glomerular diseases and are modifiable factors, control of which can slow the progression of renal disease. This is shown by a number of studies.^5^-^7^ Risk of CKD increases with three or more features of metabolic syndrome and age in diabetics as described in a study from Rawalpindi.^8^

In our study we have observed the frequency of these associated problems with glomerular diseases. At completion of one year we have simply observed the number of patients who has shown deterioration in proteinuria or renal functions while on treatment for their medical problems. This observation in rate of progression of renal disease at one year of follow up in OPD setting will increase our understanding of clinical glomerular diseases and likely to bring positive changes in our approach to manage these patients.

## METHODS

This is an observational study done at Dow University of Health Sciences, Ojha Campus Karachi. Data was collected from patients who were already on regular follow up in clinic for their glomerular disease. Patients were enrolled for the study from May 2013 till August 2013. Informed consent was taken from all patients. Patients were followed for one year from time of enrollment. Sampling was done by simple convenient method. All the adult patients attending Nephrology or Medicine OPD DUHS, with clinical glomerular disease (on history and urine detailed report showing dip stick positive proteinuria on 2 or more occasions were included in study. Exclusion criteria was age below 15 years, failure to get consent, patients with no proteinuria, patients with proteinuria along with CKD stage5 (Creatinine clearance of less than 15ml/min) at time of enrollment for study and those who were lost from follow up before completion of study. Diagnosis of clinical glomerular disease primary or secondary was made by attending physician based on presence of proteinuria with or without any systemic disease along with clinical signs and supporting laboratory reports which also includes renal biopsy done for diagnosis of primary glomerular disease. Associated problems hypertension, diabetes mellitus, dyslipidaemia, renal dysfunction, hematuria or classical nephrotic or nephritic syndrome, sarcoidosis or Systemic lupus erythromatosus were noted from case records. All concerned laboratory tests done as a routine for evaluation and follow ups were noted from case records. Serum creatinine was used to calculate creatinine clearance (Cr cl) by Cock Croft Gault formula. All the information was gathered on a proforma which was filled in person by one of researchers themselves. Data was maintained and analyzed on SPSS version 16. At the completion of follow ups laboratory reports recorded from case records which include urinary protein, and serum creatinine. Primary end point taken was completion of 1 year follow up, death or ESRF.

## RESULTS

Number of patients with clinical glomerular disease who were approached in OPDs during enrollment period was 413, out of which 324 agreed and consented to participate but follow ups were completed only on 173 who were finally included in study.

Total number of patients was 173. Mean age of patients is 51.67±10.16 (range 15 yr to 75 yr). Males were 92/173 (53.2%), females were 81/173 (46.8%), M: F ratio being 1.1: 1.0. Mean weight of our patients is 67.43+ 14.13, range 35 kg to 107 kg.

Commonest glomerular disease seen in our clinics was Diabetic Nephropathy which was found in 94.2% cases followed by lupus in 1.73%. Table-I enlist causes of glomerular diseases in study patients.

**Table I:** Primary and Secondary glomerular diseases frequencies. (n=173)

	Disease	Frequency (%)
Glomerular disease Secondary to	Diabetes Mellitus	163 (94.2)
	Lupus	03 (1.73)
	Sarcoidosis + DM	02 (1.15
	Amyloidosis	01 (0.6)
	Post streptococcal GN	1 (0.6)
Primary glomerular disease	FSGS	1 (0.6)
	Ig M Nephropathy	1 (0.6)
	Membranous Nephropathy	1 (0.6)
	Mesangiocapillary GN	1 (0.6)
	IgA Nephropathy	1 (0.6)

Among our study patients with clinical glomerular disease hypertension was found in 66.5% of patients. Renal insufficiency and dyslipidaemia was seen in more than half of patients frequencies of which are shown in Table-II.

**Table II:** Frequency of different associated problems among study patients.(n=173)

	Yes(%)	No(%)	Not known(%)
Diabetes mellitus	163 (94.2)	10 (5.78)	00
Hypertension	115 (66.5)	58 (33.5)	00
Renal insufficiency	98 (56.6)	75 (43.4)	00
hypertiglyceredemia	85 (49.1)	83 (48)	5 (2.9)
Hypercholesterolemia	72 (41.6)	96 (55.5)	5 (2.9)
Clinical Nephrotic syndrome	4 (2.3)	169 (97.7)	00
Microscopic hematuria	2(1.2)	171 (98.8)	00

Evaluation at completion of one year follow up are shown in Table-III. There was no change found in grades of proteinuria in our patients. There were 3 patients with nephrotic syndrome at start of study and the frequency was same at one year follow up.

**Table III:** Results of Cr cl and proteinuria on completion of one year.

Cr clearance (ml/min)	At start of study	At six months	At one year
	N=173(%)	N=141(%)	N=137(%)
>90	81 (46.8)	76 53.9)	72 (52.5)
60-89	51(29.5)	30 (21.2)	31 (22.6)
30-59	30(17.3)	21 (14.8)	20 (14.6)
15-29	11(6.4)	11(7.8)	10 (7.3)
<15	0	3 92.1)	4 (2.9)
Not availabel	0	32	36

Proteinuria grad	At start of study	At six mnths	At one year
	N=173(%)	N=173(%)	N=172(%)
negative	0	0	1
<1 gm/d	156 (90.2)	153 (88.4)	153 (88.9)
1-3.5 gm/d	14 (8.1)	17(9.8)	15(8.7)
>3.5 gm/d	3 (1.7)	3(1.7)	3(1.74)
Not availabel	0	0	1

Only one patient shows negative proteinuria on dipstick at one year of follow up, to begin with this patient had less than 1 gm of proteinuria. Stage 5 CKD with Cr cl of less than 15ml/min was found in 3 (2.1%) patients at 6 months and in 4 (2.9%) patients at one year follow up. All of these patients on further evaluation were found to proceed from stage 4 (Cr cl of 30-15 ml/min) to stage 5 (Cr cl of less than 15ml/min) over one year. No death was noted among our study patients during this one year follow up period.

## DISCUSSION

Glomerular diseases are an important cause of morbidity and mortality. Primary glomerular diseases are diagnosed based on renal biopsy findings. Most of secondary glomerular diseases are diagnosed clinically in the presence of systemic disease and associated renal involvement as evaluated by simple lab tests. Biopsy is required for diagnosis of secondary glomerular diseases only if diagnosis is in doubt or to decide for immunosuppressive treatment.

We found that secondary glomerular diseases are far more common (98%) than primary glomerular diseases. Most of patients included in our study were diagnosed clinically by physicians and renal biopsies were required and performed in only ten patients.

Epidemiological data from China on biopsy proven cases of glomerular disease have shown primary glomerular diseases are common than secondary glomerular diseases.^9^ Similar results are also found in other studies which uses renal biopsies for diagnosis of glomerular diseases all showing that primary glomerular diseases are more common than secondary glomerular diseases.^3^,^4^

The difference observed in pattern of primary and secondary diseases frequencies between our study and other studies can easily be explained on basis of method adopted for diagnosis of glomerular diseases being clinical in our patients and biopsy in other studies. Almost all of our patients are Asian and Pakistani so ethnicity risk is similar in all. We observed no obvious gender discrimination.^10^

If we look at associated medical problems in patients with glomerular diseases we found that diabetes, hypertension, hypertriglyceridemia and renal insufficiency are very common accompaniment of glomerular diseases. All these illness increases risk of cardiovascular disease in patients with renal disease and is well documented in literature that CKD early diagnosis and intervention can prevent or delay end stage renal failure.^11^,^12^

Number of studies have tried to see outcome in glomerular diseases and also to identify modifiable risk factors to improve prognosis.^13^ One study done on 497 patients followed for 9 years is showing an improved prognosis and survival in patients with type 1 diabetic nephropathy.^14^ Antihypertensives and Lipid Lowering Treatment to Prevent Heart Attack Trial (ALLHAT) has shown protective role of anti hypertensives and lipid lowering agents in progression of cardiovascular disease and end stage renal failure.^6^

Dyslipidemia is considered an important modifiable risk in progression of cardiovascular and chronic kidney disease. A number of studies have been done to show statins protective role in chronic kidney disease. Protective role of statins is well documented in subgroups of patients with chronic kidney disease.^7^,^15^ Other factors like body mass index and ethnicity are also influencing course of diabetic nephropathy.^16^-^18^

A number of factors affect outcome in glomerular diseases like familial FSGS carry poor prognosis than sporadic FSGS.^19^ Treatment failure in Lupus is a known risk factor for progression to end stage renal failure.^20^

Evaluation of our results at completion of one year have shown no change in grades of proteinuria. There were three patients with nephrotic syndrome at start of study and the frequency was same at one year follow up. Only one patient shows negative proteinuria on dipstick at one year of follow up, to begin with this patient had less than 1 gm of proteinuria.

Stage 5 CKD with creatinine clearance of less than 15ml/min was found in 3 (2.1%) patients at 6 months and in 4 (2.9%) patients at one year follow up. Only one among them required hemodialysis. All of these patients on further evaluation were found to proceed from stage 4 to stage 5 over one year. No death was noted among our study patients during this one year follow up period.

Hematuria with glomerular diseases seen in only 1.2% of our studied population, likely because most of our patients were with diabetic nephropathy and hypertensive sclerosis of which hematuria is not a feature. Progression of CKD from stage 3 to 5 is predicted by male gender, diabetes, low hematocrit, high systolic B.P and low albumin.^21^

Proper control of blood glucose levels, blood pressure and lipid profiles have already shown to be associated with regression or remission of microalbuminuria.^22^-^24^ All of our patients with diagnosis of diabetes and proteinuria were started on conservative treatment with good control of diabetes and hypertension preferably using angiotensin converting enzyme inhibitors or receptor blockers.

Evaluation for creatinine clearance at one year follow up was deficient in 36 patients which might have screwed interpretation of results. However overall results are not demonstrating any remarkable change in Cr cl of patients with levels more than 30ml/minutes. Only important change noted was progression to ESRF in 4 /137 (2.9%) patients which on detailed observation were found to be those who were already in stage 4. This indicates that one year follow-up in patients with stage 4 renal failure is important as there is a likelihood of these patients progression to stage 5. Follow up of these patients should be close at frequent intervals with early planning and attainments of permanent angio-access as a preparation for renal replacement therapy.

### Limitations of study

Based on easy availability we selected our patients on dipstick positivity and patients with microalbuminuria might be missed when negative for ordinary urinary dipstick test. Missing values for one patient at one year for urinary proteins and 36 patients for serum creatinine might have altered our overall interpretation.

## CONCLUSION

Secondary glomerular diseases are far more common than primary glomerular diseases in clinical practice, diabetic nephropathy being the commonest. At one year follow up no change in proteinuria level was found. One year follow up is critical for patients of glomerular disease with stage 4 CKD as progression to end stage renal failure may seen within one year in these patients.
